# Deletion of Fn14 receptor protects from right heart fibrosis and dysfunction

**DOI:** 10.1007/s00395-012-0325-x

**Published:** 2013-01-17

**Authors:** Tatyana Novoyatleva, Yves Schymura, Wiebke Janssen, Frederic Strobl, Jakub M. Swiercz, Chinmoy Patra, Guido Posern, Astrid Wietelmann, Timothy S. Zheng, Ralph T. Schermuly, Felix B. Engel

**Affiliations:** 1 Department of Cardiac Development and Remodelling, Max Planck Institute for Heart and Lung Research, Parkstrasse 1, 61231 Bad Nauheim, Germany; 2 Department of Lung Development and Remodelling, Max Planck Institute for Heart and Lung Research, Parkstrasse 1, 61231 Bad Nauheim, Germany; 3 Department of Pharmacology, Max Planck Institute for Heart and Lung Research, Parkstrasse 1, 61231 Bad Nauheim, Germany; 4 Institut für Physiologische Chemie, University Halle-Wittenberg, Hollystrasse 1, 06114 Halle, Germany; 5 Nuclear Magnetic Resonance Imaging, Max Planck Institute for Heart and Lung Research, Parkstrasse 1, 61231 Bad Nauheim, Germany; 6 R&D, Immunology, Biogen Idec, Inc., Cambridge, MA 02142 USA; 7 Department of Pulmonary Pharmacotherapy, Justus-Liebig-University Giessen, Aulweg 130, 35392 Giessen, Germany; 8 Experimental Renal and Cardiovascular Research, Department of Nephropathology, Institute of Pathology, University of Erlangen-Nürnberg, Universitätsstraße 22, 91054 Erlangen, Germany

**Keywords:** Right heart disease, Fibrosis, Fn14, MAL, Cardiac fibroblasts

## Abstract

Pulmonary arterial hypertension (PAH) is a fatal disease for which no cure is yet available. The leading cause of death in PAH is right ventricular (RV) failure. Previously, the TNF receptor superfamily member fibroblast growth factor-inducible molecule 14 (Fn14) has been associated with different fibrotic diseases. However, so far there is no study demonstrating a causal role for endogenous Fn14 signaling in RV or LV heart disease. The purpose of this study was to determine whether global ablation of Fn14 prevents RV fibrosis and remodeling improving heart function. Here, we provide evidence for a causative role of Fn14 in pulmonary artery banding (PAB)-induced RV fibrosis and dysfunction in mice. Fn14 expression was increased in the RV after PAB. Mice lacking Fn14 (Fn14^−/−^) displayed substantially reduced RV fibrosis and dysfunction following PAB compared to wild-type littermates. Cell culture experiments demonstrated that activation of Fn14 induces collagen expression via RhoA-dependent nuclear translocation of myocardin-related transcription factor-A (MRTF-A)/MAL. Furthermore, activation of Fn14 in vitro caused fibroblast proliferation and myofibroblast differentiation, which corresponds to suppression of PAB-induced RV fibrosis in Fn14^−/−^ mice. Moreover, our findings suggest that Fn14 expression is regulated by endothelin-1 (ET-1) in cardiac fibroblasts. We conclude that Fn14 is an endogenous key regulator in cardiac fibrosis and suggest this receptor as potential new target for therapeutic interventions in heart failure.

**Electronic supplementary material:**

The online version of this article (doi:10.1007/s00395-012-0325-x) contains supplementary material, which is available to authorized users.

## Introduction

Pulmonary arterial hypertension is a fatal disease with a 3-year mortality rate of 20–40 % for which no cure is available [12, 18, 43]. During disease progression, the RV undergoes compensatory hypertrophy to maintain physiological blood pressure and flow. As PAH progresses the RV becomes fibrotic and dilates and ultimately undergoes functional failure [8, 38]. In recent years, the outlook for PAH patients has improved due to earlier recognition and new therapies. However, despite these interventions vascular pulmonary resistance was high and increased over time leading eventually still to RV failure [8, 41]. Thus, it is important to elucidate mechanisms driving RV remodeling and transition to RV dysfunction to identify new targets for therapeutic intervention [47].

Previously, the receptor Fn14 and its ligand TWEAK have been associated with LV remodeling after myocardial infarction (MI) [6, 33]. Systemic overexpression of TWEAK induced via Fn14 progressive dilated cardiomyopathy and heart failure affecting both the LV and RV [20]. This phenotype was associated with cardiomyocyte elongation and cardiac fibrosis. Fn14 is also expressed in human cardiomyocytes and circulating TWEAK levels were correlated with idiopathic dilated cardiomyopathy [20]. In contrast, TWEAK levels were inversely correlated with the severity of PAH in patients suggesting that the TWEAK/Fn14 axis might play no role in the RV failure [11]. However, correlations of cytokine blood levels to heart disease can be misleading. For example, TNF overexpression leads to heart failure and its endogenous expression is positively correlated with heart failure [23]. However, clinical trials with anti-TNF therapies were disappointing [27]. Moreover, as with PAH, TWEAK blood plasma levels are decreased in patients and animal models with chronic kidney disease (CKD). In contrast, Fn14 is upregulated in the kidney in animal models as well as patients with CKD [19, 52]. Deletion of Fn14 in the animal models protects against kidney fibrosis and failure [19]. Thus, it is important to study the endogenous role of Fn14 in heart disease.

TWEAK plays an important role in several biological processes including inflammation, angiogenesis, cell growth, cell death and fibrosis. Depending on cell type and context TWEAK mediates these activities via Fn14 by activating a variety of downstream signaling cascades. Our previous data have suggested that TWEAK-induced cardiomyocyte proliferation is mediated through activation of ERK and PI3K as well as inhibition of GSK-3beta [35]. Recently, NFκB has been identified as a major downstream target of the TWEAK/Fn14 axis through functional validation in numerous cellular and invivo contexts [31]. With regard to signaling in heart cell types, the TWEAK/Fn14 axis was shown to induce NFκB signaling in cardiomyocytes [6] as well as fibroblasts [5].

Here we examined Fn14 expression in models of pressure overload (3 weeks after PAB in mice [2, 3] or monocrotaline (MCT) treatment of rats [7, 26]) and found Fn14 markedly upregulated in the RV. Fn14^−/−^ mice exhibited reduced fibrosis and improved RV function following PAB. Our data show that Fn14 activation regulates fibroblast proliferation, differentiation and collagen expression. Finally, our data suggest a novel TWEAK/Fn14/RhoA/MAL pathway downstream of ET-1 signaling in cardiac fibroblasts (CFs). Collectively, our data demonstrate that Fn14 is an important endogenous mediator of RV remodeling and failure.

## Methods

An expanded version of methods is available in Supplementary material online.

### Animal studies

The investigation conforms with the Guide for the Care and Use of Laboratory Animals published by the Directive 2010/63/EU of the European Parliament. In vivo procedures were approved by a local Animal Ethics Committee in accordance to governmental and international guidelines on animal experimentation. Fn14^−/−^ mice were previously generated at Biogen (Biogen Idec, Inc, Cambridge) [21]. PAB and/or SHAM operation of mice (20–23 g) was performed under isoflurane anesthesia (1.5 % v/v) and 0.03 mg/kg buprenorphine hydrochloride (s.c.). Analgetic therapy post operation was achieved by buprenorphine (0.03 mg/kg, 48 h) and carprofen (s.c., 4 mg/kg, 3–7 days). Pulmonary hypertension in Sprague–Dawley rats (300–350 g) was induced with 60 mg/kg MCT (s.c.) [42]. Animals were daily controlled for signs of pain. Functional analyses were performed by MRI and hemodynamic measurements with a Millar microtip catheter under inhalation of isoflurane (1.5–2.0 % v/v). For organ/tissue sampling, animals were anesthetized (120 mg/kg ketamine + 16 mg/kg xylazine) and subsequently euthanized through exsanguination. Blood was intracardially collected and analyzed with a RayBio_Mouse TWEAK ELISA Kit.

### Cell culture experiments

Cardiac fibroblasts were isolated with Liberase TH enzyme mix (Roche) from Fn14^−/−^ mice and wild-type littermates. All experiments were performed with primary CFs after one or two passages. As an immortalized fibroblast cell line the Rat2 fibroblast cell line was used; a normal, non-tumorigenic and highly transfectable cell line derived from the Fischer rat fibroblast 3T3 like cell line Rat1. HEK293T cells were utilized for luciferase reporter assays. NIH3T3 cells were used as control cells for MAL nuclear translocation assays. Cells were cultured under standard conditions or as indicated.

### Construction of pEGFP-TWEAK

TWEAK cDNA was amplified from HEK293T cells, ligated into pGEM-T-easy and a *Hind*III/*Sal*I TWEAK fragment was subcloned into pEGFP-N1.

### Transfection and luciferase promoter assays

Transient transfections were performed with Fugene 6 (Roche) with a total of 50 ng of constructs. Luciferase activity was measured by LightSwitch Luciferase Assay Reagent after 24 h of stimulation or 30–48 h after overexpression.

### Western blot

Tissues were lysed and homogenized in RIPA buffer. Protein extracts were resolved on SDS gels, blotted on nitrocellulose membranes and incubated with the following primary antibodies: rabbit anti-TWEAK/Fn14 receptor, rabbit anti-pan-actin, monoclonal rabbit anti-RhoA (1:1,000) (all cell signaling), goat anti-DDR2 (Santa Cruz), rabbit anti-Collagen Type 1 (1:500) (Rockland Immunochemicals), mouse anti-GAPDH (1:2,000) (Sigma) and polyclonal rabbit anti-MAL (1:200) (from G. Posern). Antigen–antibody complexes were visualized using horseradish peroxidase-conjugated secondary antibodies. Western blots were quantified by Image J (NIH) software.

### siRNA-mediated knockdown of MAL

Cardiac fibroblasts from wild-type mice were split in serum-free medium and 24 h later transfected with 40 pM of siRNA perfectly matching the sequence 5′-ATGGAGCTGGTGGAGAAGAA-3′ of both murine MAL and MRTF-B and 40 pM of scrambled siRNA (Qiagen) with lipofectamine [30]. 24 h later cells were stimulated with 100 ng/ml of TWEAK.

### Determination of activated RhoA

Activated RhoA was determined by immunoprecipitation with the fusion protein GST-Rho-binding domain of Rhotekin (GST-RBD) [39].

### RNA extraction and Real-Time PCR

RNA was isolated with an RNeasy Fibrous Tissue Kit. RT reaction was performed using oligo (dT) primer. For Real-Time PCR analysis, cDNA was amplified with IQTM SYBR^®^ Green SuperMix (Biorad) and Bio-Rad iCYCLER iQ5. Real-Time PCR was performed in triplicates and relative gene expression was calculated on the basis of ΔCt values to *gapdh*.

### Immunohistochemistry

Mouse hearts were isolated, dissected in RV and LV + S, washed in PBS, fixated overnight in 10 % PFA, embedded in paraffin and sectioned longitudinally (5 μm). Sections were deparaffinized in xylene and rehydrated in ethanol. Heat-mediated antigen retrieval was performed in 0.05 M EDTA buffer (pH 8.0). Tissues for cryosections (5 μm) were frozen in OCT, fixated in acetone and stained as indicated. To quantify fibrosis RV sections were stained with 0.1 % Sirius Red F3B in picric acid and analyzed using a QWin V3 computer-assisted image analysis software (Leica) [15].

### Immunofluorescence

Heart sections were blocked in 5 % goat serum/0.2 % Tween-20/PBS for 1 h at RT. Cells were fixated in 3.7 % paraformaldehyde and permeabilized in PBS/0.5 % Triton. Samples were stained as indicated. F-actin was detected by rhodamine–phalloidin and cell membranes by WGA staining (Molecular Probes, Invitrogen). Cell size was determined using the ImageJ (NIH) software.

### Endothelin stimulation

After serum starvation cells were stimulated for 24 h with 100 nM ET-1 (R and D systems).

### Collagen assay

Serum-starved Rat2 fibroblasts were stimulated with TWEAK (100 ng/ml) for 48 h. l-ascorbic acid (0.25 mM) was added to the medium daily. Cells were lysed in RIPA buffer and total collagen (Types 1–5) was assessed using a Sircol soluble collagen assay kit (Biocolor Ltd).

### MAL translocation

MAL translocation was determined with the Count software (by B. Waclaw). Nuclei number was assessed based on DAPI staining. MAL translocation was considered positive if the pixel number in the purple channel (MAL/DAPI overlap) was greater or equal compared to the threshold defined by control experiments.

### Proliferation assay

Proliferation was determined with a Countess™ cell counter (Invitrogen) or CellTiter 96 Aqueous Cell Proliferation Assay (Promega).

### Statistical analyses

Data were analyzed with GraphPad Prism. Data are presented as mean ± standard error of the mean (SEM). Statistical significance was determined using Student’s *t* test or for multiple comparisons One-way ANOVA followed by Bonferroni’s post hoc test. Values of *p* < 0.05 were considered statistically significant.

## Results

### Fn14 expression is upregulated in models of RV failure

**Fig. 1:**
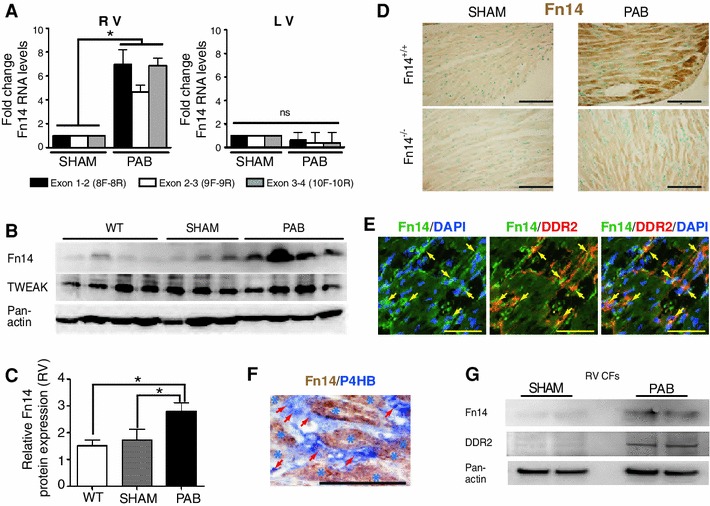
Fn14 expression in RV heart disease. **a** Real-Time PCR analyses of Fn14 mRNA expression from RV demonstrated elevated expression following PAB. No significant changes were observed in LVs (*p < 0.05, *n* = 4). Loading control: *gapdh*. **b** Western blot analysis of RV extracts of WT, SHAM- and PAB-operated mice showing a prevalent expression of Fn14 after PAB in RV. No significant difference was observed for TWEAK. Loading control: Pan-actin. **c** Quantitative analysis of **b**. Fn14 levels (normalized to pan-actin) of four individual hearts per condition were expressed as arbitrary units ± SEM. **d** Immunohistochemistry: Fn14 protein expression is elevated after PAB in RV of Fn14^+/+^ animals. Nuclei were counterstained with methyl green. Sections from Fn14^−/−^ mice served as control for the specificity of the used anti-Fn14 antibody. **e** and **f** Co-staining experiments with fibroblast-markers (**e**, DDR2; **f**, P4HB: prolyl 4-hydroxylase, beta polypeptide) indicating that Fn14 expression is upregulated in fibroblasts after PAB in RV of Fn14^+/+^ animals. *Arrows*: Fn14-positive fibroblasts. *Stars*: cardiomyocytes. **g** Western blot analysis: Fn14 is upregulated in isolated Fn14^+/+^ CFs from RVs after PAB. *Scale bars*: 65 μm. *RV* right ventricle, *LV* left ventricle, *PAB* pulmonary artery banding

To assess whether Fn14 signaling is involved in RV failure, we determined the expression levels of Fn14 in RVs 3 weeks after PAB in mice by Real-Time PCR. LVs were used as an internal control as they are not affected in this model. Fn14 mRNA expression was not upregulated in LVs (Fig. 1a). In contrast, Real-Time PCR analyses utilizing primers spanning the TWEAK-binding (exon 1–2), transmembrane (exon 2–3, only present in full-length) and TRAF-binding cytosolic (exon 3–4) motifs of Fn14 demonstrated upregulated expression of full-length Fn14 (NM_013749.2) in RVs of mice after PAB (Fig. 1a). A correlation between RV dysfunction and Fn14 expression was confirmed at protein level after PAB (Fig. 1b, c). In contrast to TWEAK, Fn14 was strongly expressed throughout the diseased RV (Fig. 1d and Supplemental Fig. 1a). Co-staining experiments indicated that Fn14 expression is upregulated in cardiomyocytes and fibroblasts (Fig. 1e, f). This was further supported by Western blot analyses of RV mouse CFs after PAB (Fig. 1g). Taken together our data suggest a positive correlation between upregulation of Fn14 in the heart and RV failure.

Previously, it has been determined that TWEAK levels are inversely correlated with the severity of PAH in patients. This observation led to the conclusion that the TWEAK/Fn14 axis may have no role in RV disease [11]. In contrast to the observation in humans, TWEAK levels were unchanged in PAB-operated animals (Supplemental Fig. 1b). To further investigate this controversy we utilized a second RV disease model MCT treatment in rats. Neither Fn14 nor TWEAK mRNA expression was upregulated in LVs. In contrast, Fn14 and TWEAK mRNA expression were markedly upregulated in RVs after MCT treatment (Supplemental Fig. 1c, d). In contrast, TWEAK blood plasma levels were significantly reduced (Supplemental Fig. 1e). Thus, TWEAK blood plasma levels do not necessarily correlate with the expression levels of TWEAK and/or Fn14 in the heart.

### Fn14^−/−^ mice are resistant to PAB-induced RV dysfunction

**Fig. 2:**
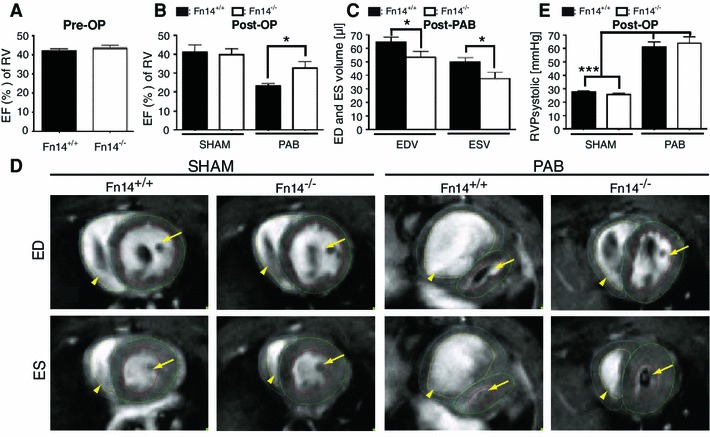
Fn14^−/−^ mice show improved heart function after PAB. MRI imaging. **a** RV ejection fraction (EF) before PAB was not significantly different between wild-type and knockout mice (SHAM: Fn14^+/+^: *n* = 4; Fn14^−/−^: *n* = 6; PAB-operated mice: *n* = 11 both for Fn14^+/+^ and Fn14^−/−^). **b** RV EF was markedly decreased 3 weeks after PAB in Fn14^+/+^ mice (*n* = 11, ***p < 0.0001). Reduction of RV EF was significantly inhibited in Fn14^−/−^ mice (*n* ≥ 4 for SHAM-operated mice; *n* = 11 for PAB-operated mice, *p < 0.05). **c** End-diastolic (ESV) and EDVs (*p < 0.05, *n* = 11 for PAB-operated mice). **d** Representative MRI images of hearts from SHAM- and PAB-operated mice. *ED* end-diastole, *ES* end-systole. **e** Peak of RV systolic pressure. *RV* right ventricle, *PAB* pulmonary artery banding. LVs are indicated by *arrows*, RVs by *arrowheads*

To evaluate a causative role of Fn14 upregulation and RV failure, we challenged wild-type Fn14^+/+^ and knockout Fn14^−/−^ mice with PAB and analyzed RV function. Under physiological conditions, no significant differences were observed. A consistent increase in systolic RV pressure in Fn14^+/+^ as well as Fn14^−/−^ PAB-operated mice confirmed proper banding (Fig. 2e). After PAB, Fn14^+/+^ mice exhibited dramatic increases in RV end-systolic volume (ESV) and end-diastolic volume (EDV), indicating dilation and a decrease in RV ejection fraction (EF). In contrast, Fn14^−/−^ mice were resistant to RV dilation showing a significantly better RV EF after PAB (Fig. 2 and Supplemental Table S1). In conclusion, these results indicate that Fn14 is a potent endogenous mediator of RV dysfunction and that deletion of Fn14 protects mice from PAB-induced RV dysfunction.

### Fn14 signaling regulates fibrosis

**Fig. 3:**
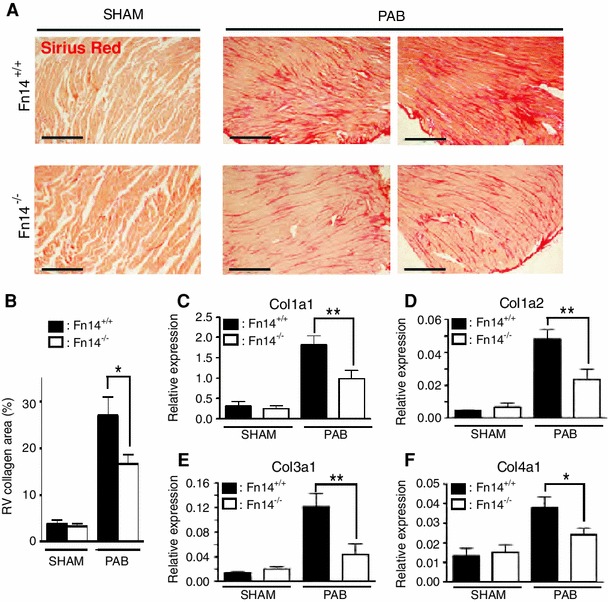
Fn14 ablation reduces myocardial fibrosis after PAB. **a** Sirius Red staining demonstrating attenuation of RV fibrosis 3 weeks after PAB in Fn14^−/−^ mice. *Scale bars*: 300 μm. **b** Quantitative analysis (SHAM: Fn14^+/+^ and Fn14^−/−^: *n* = 4, PAB: Fn14^+/+^: *n* = 5, Fn14^−/−^: *n* = 7, *p < 0.05). **c–f** Real-Time PCR demonstrating that upregulation of Col1a1, Col1a2, Col3a1 and Col4a1 expression in RVs after PAB was diminished in Fn14^−/−^ mice. Expression levels were normalized to *gapdh* (**p < 0.005, *p < 0.05, *n* = 4). *RV* right ventricle, *PAB* pulmonary artery banding

Three weeks post-PAB, the fibrotic area was reduced by 39 % fibrosis in RVs of Fn14^−/−^ mice compared to Fn14^+/+^ littermates (Fig. 3a, b). Moreover, PAB-induced upregulation of fibrosis-associated collagen genes like Col1a1 and Col1a2 was diminished (Fig. 3c–e). Expression levels of collagens were not affected in the LV (data not shown). These data suggest that Fn14 is an endogenous mediator of fibrosis in RV heart disease.

### Fn14 signaling regulates collagen expression via the RhoA-Mal axis

**Fig. 4:**
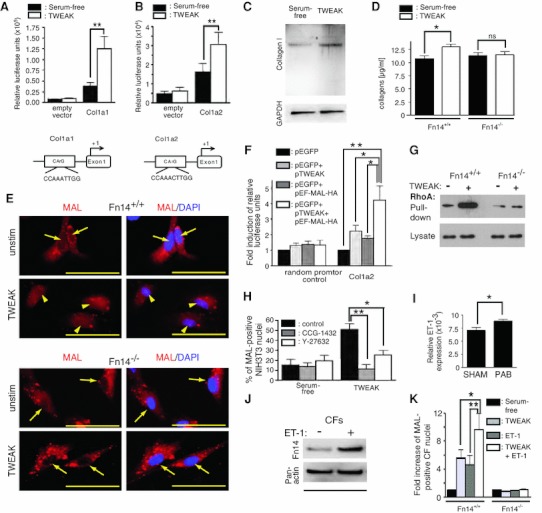
Fn14 signaling regulates collagen expression via the RhoA-Mal axis. (**a** and **b**) Luciferase reporter assays reveal that TWEAK stimulation activates Col1a1 and Col1a2 promoter (*n* = 7, **p < 0.01). HEK293T cells were transfected with indicated promoter constructs and 24 h later stimulated with TWEAK. **c** Western blot analysis of TWEAK-stimulated Rat2 fibroblasts showing markedly upregulated Collagen I expression. **d** TWEAK-induced production of collagens in CFs depends on Fn14 (*n* = 4, TWEAK versus serum-free, Fn14^+/+^: *p < 0.05; Fn14^−/−^: not significant). **e** TWEAK-induced nuclear translocation of MAL in cardiac fibroblasts depends on Fn14. *Arrows* indicate MAL-negative nuclei. *Arrowheads* indicate MAL-positive nuclei. *Scale bars*: 50 μm. **f** Promoter assays in HEK293T revealed enhancement of Col1a2 promoter activation after MAL and TWEAK overexpression. pEGFP-N1 (pEGFP) was used as control plasmid. **g** Amount of activated RhoA determined by immunoprecipitation with Rho-binding domain of Rhotekin (GST-RBD) in Fn14^+/+^ and Fn14^−/−^ CFs. **h** Pre-incubation of NIH3T3 cells with ROCK kinase inhibitor Y27632 (20 μM) [*p < 0.05 vs. DMSO (control)] and CCG-1432 (5 μM) [**p < 0.005 vs. DMSO (control)] inhibited TWEAK-induced nuclear MAL translocation (*n* = 3). *RV* right ventricle, *PAB* pulmonary artery banding. (*I*) Real-Time PCR revealed a significant increase in ET-1 levels in RVs following PAB in wildtype Fn14^+/+^ mice. Loading control: *gapdh* (*n* > 3, *p < 0.05). **j** Western blot analysis: ET-1 (100 nM) induces Fn14 expression in Fn14^+/+^ CFs (*n* = 3). **k** Co-stimulation with TWEAK/ET-1 markedly induced MAL translocation in Fn14^+/+^ CFs compared to ET-1 (*p < 0.01) and TWEAK stimulation alone (*p < 0.05) (four mice per each group, *n* = 4)

To further understand Fn14 signaling with regards to fibrosis, we utilized cell lines and primary CFs expressing Fn14 endogenously (Fig. 4 and Supplemental Fig. 2a). HEK293T cells were utilized as standard cell type for luciferase promoter assays. TWEAK treatment of HEK293T cells markedly enhanced Col1a1 and Col1a2 promoter activity (Fig. 4a, b). To determine collagen synthesis, we utilized the Rat2 fibroblast cell line, as HEK293T are suboptimal for such studies [34]. TWEAK treatment of Rat2 fibroblasts resulted in the accumulation of collagens, which was abolished by co-treatment with ITEM2, an Fn14 blocking antibody (Fig. 4c and Supplemental Fig. 2b, c). Importantly, TWEAK treatment also enhanced collagen expression by primary CFs of Fn14^+/+^ mice, but had no effect on CFs of Fn14^−/−^ mice (Fig. 4d). Collectively, we conclude that Fn14 regulates collagen expression in fibroblasts.

It has been demonstrated that the transcription factor MAL plays a regulatory role in collagen expression. Furthermore, it has been shown that the Col1a2 promoter is a direct target of MAL [44] and that serum stimulation of NIH3T3 cells induces MAL nuclear translocation [32]. To determine if the TWEAK/Fn14 axis promotes MAL nuclear translocation NIH3T3 cells and primary Fn14^+/+^ CFs were stimulated with TWEAK. As a positive control we used 15 % serum. Stimulation of Fn14^+/+^ CFs with TWEAK resulted in nuclear translocation of MAL (Fig. 4e and Supplemental Fig. 3a, b), which was inhibited in Fn14^−/−^ CFs (Fig. 4e). In addition, MAL overexpression enhanced TWEAK-induced Col1a2 promoter activity in HEK293T cells (Fig. 4f). Finally, siRNA-mediated knockdown of MAL abolished TWEAK-induced collagen expression (Supplemental Fig. 2d, e). These results suggest that Fn14 activation promotes collagen expression via induction of MAL nuclear translocation.

MAL is regulated through the Rho GTPase-actin pathway [32]. Activation of RhoA determined by a Rhotekin pull down assay was first established in HEK293 cells (Supplemental Fig. 3c). TWEAK stimulation of primary Fn14^+/+^ CFs but not of Fn14^−/−^ CFs led to strong RhoA activation (Fig. 4g). Moreover, MAL translocation to the nucleus could be suppressed by inhibitors to ROCK kinase (Y27632) or the Rho/SRF pathway (CCG-1432) in NIH3T3 cells (Fig. 4h). These results indicate that TWEAK can promote MAL translocation via RhoA activation.

### ET-1 regulates Fn14

ET-1 is an important player in the pathogenesis of PAH. Upregulation of ET-1 in patients is associated to PAH [46] and cardiac fibrosis [36]. Its blood level is upregulated after PAB in mice [13, 37] and in PAH patients [14]. Thus, we suspected that ET-1 might be responsible for the upregulation of Fn14 upon PAB. As hypothesized, PAB resulted in the upregulation of ET-1 in wildtype Fn14^+/+^ mice (Fig. 4i). Importantly, exposure of primary CFs to ET-1 resulted in marked upregulation of Fn14 expression (Fig. 4j). Finally, ET-1 stimulation significantly enhanced TWEAK-induced nuclear MAL translocation in Fn14^+/+^ CFs, but not Fn14^−/−^ CFs (Fig. 4k). In conclusion, our data suggest that ET-1 enhances signaling through the Fn14-RhoA-MAL axis inducing collagen expression and thereby promoting RV fibrosis.

### Fn14 signaling promotes myofibroblast differentiation

**Fig. 5:**
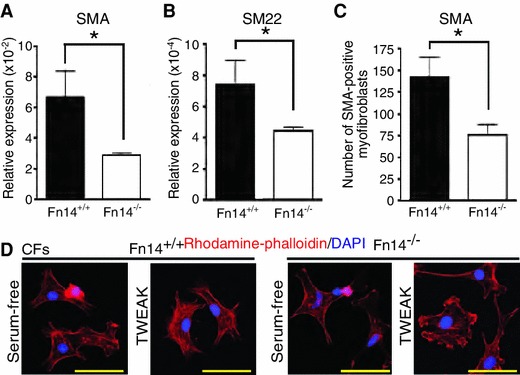
Fn14 regulates myofibroblast differentiation. (**a** and **b**) Real-Time PCR revealed a significant reduction of SM22 and SMA induction in RVs of Fn14^−/−^ PAB-operated mice. Loading control: *gapdh* (*n* = 4, *p < 0.05). **c** Quantification of SMA-positive myofibroblasts (four mice per group, *p < 0.05). **d** TWEAK stimulation resulted in the enrichment of actin fibers (Rhodamine-phalloidin, *red*) in Fn14^+/+^, but not Fn14^−/−^ CFs. *Scale bars*: 50 μm

Another characteristic upon tissue injury besides collagen expression is the differentiation of interstitial fibroblasts into myofibroblasts, which are characterized by the expression of smooth muscle cell (SMC) markers. As the SRF co-activator MAL is also critical for the induction of the myofibroblast-associated genes α-smooth muscle actin (SMA) and α-smooth muscle protein 22 (SM22), we analyzed Fn14^−/−^ mice for defects on myofibroblast differentiation [44]. Expression of SMA and SM22 was attenuated in RVs of Fn14^−/−^ animals after PAB (Fig. 5a, b). However, as SMA is expressed in spindle-shaped myofibroblasts as well as smooth muscle cells in blood vessels, we performed immunofluorescence stainings. We detected in Fn14^−/−^ RVs significantly lower numbers of spindle-shaped SMA-positive myofibroblasts, which were not associated with any vessel (Fig. 5c). Importantly, TWEAK stimulation induced the accumulation of organized stress fibers, a hallmark of myofibroblasts, in Rat2 fibroblasts (Supplemental Fig. 3d) as well as in Fn14^+/+^ CFs, but not Fn14^−/−^ CFs (Fig. 5d). These data identify Fn14 as a potential mediator of myofibroblast differentiation.

### Fn14 regulates fibroblast proliferation

**Fig. 6:**
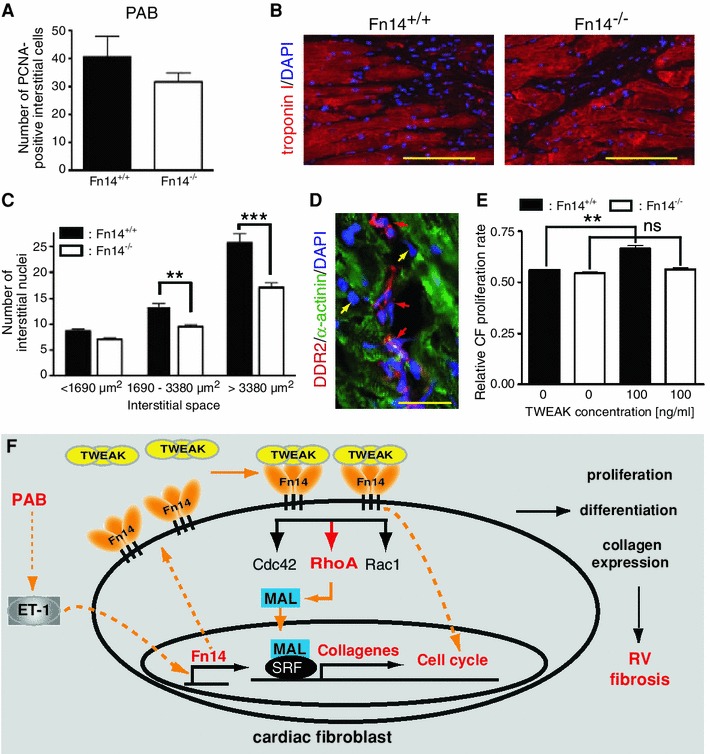
Fn14 regulates fibroblasts proliferation. **a** Quantitative analysis of PCNA-positive interstitial cells after PAB (Fn14^+/+^: 6 and, Fn14^−/−^: seven mice per group). **b** Representative examples of stained heart sections used to determine interstitial nuclear density. *Scale bars*: 100 μm. **c** Quantification of **b** (Fn14^+/+^: six mice and Fn14^−/−^: seven mice per group, **p < 0.005, ***p < 0.0001). **d** Immunofluorescence analyses: the majority of cells in the clusters are fibroblasts (DDR2). *Red arrow*: fibroblasts. *Yellow arrows*: non-fibroblast interstitial cells. *Scale bars*: 25 μm. **e** TWEAK increased the number of Fn14^+/+^, but not Fn14^−/−^ CFs in a dosage-dependent manner (*n* = 5, **p < 0.001). **f** Proposed model of TWEAK/Fn14 axis activity during RV fibrosis. *RV* right ventricle, *PAB* pulmonary artery banding

Enhanced fibroblast proliferation can also contribute to alteration of connective tissue homeostasis and fibrosis under pathophysiological conditions [44]. Quantification of staining of the proliferation marker proliferating cell nuclear antigen (PCNA) did not indicate a change in cardiomyocyte proliferation after PAB (data not shown). There was, however, a trend towards decreased proliferation of interstitial cells corroborating with a diminished interstitial cell density in Fn14^−/−^ hearts (Fig. 6a, b). Quantification showed that the total number of interstitial cells was significantly higher in RVs from Fn14^+/+^ animals compared to Fn14^−/−^ littermates (Fig. 6c). Immunofluorescence analyses with the fibroblast-specific marker discoidin domain receptor 2 (DDR2) revealed that the major cell type in the interstitial cell clusters is fibroblasts (Fig. 6d). These data suggest that fibroblast proliferation was decreased in the absence of Fn14.

To directly address whether TWEAK/Fn14 signaling is sufficient to induce fibroblast proliferation, Rat2 cells and CFs (>90 % fibroblasts, Supplemental Fig. 4a) were stimulated with TWEAK in serum-free medium. TWEAK stimulation induced Rat2 fibroblast proliferation in a concentration-dependent manner. Fn14 overexpression by itself resulted in proliferation in serum low conditions (Supplemental Fig. 4b–d). Importantly, in vitro TWEAK stimulation of CFs enhanced proliferation of Fn14^+/+^ but not of Fn14^−/−^ CFs (Fig. 6e) supporting the notion that the TWEAK/Fn14 axis regulates fibroblast proliferation.

Taken together, these results indicate that the protective effect of Fn14 deletion could be explained in part by the impact of Fn14 activation on fibroblast proliferation, differentiation and collagen expression (Fig. 6f).

## Discussion

The development of RV failure involves complex pathological mechanisms whereas identification of underlying causes and successful medical treatment remain a major challenge. Here, we show that Fn14 deletion reduces markedly PAB-induced right heart fibrosis and dysfunction. Our data provide strong evidence that inhibition of endogenous TWEAK/Fn14 pathway may potentially be clinically beneficial in treating right heart disease due to pressure overload. This is important as right heart disease is poorly characterized with limited treatment options. Furthermore, our study suggests that Fn14 is linked at the mechanistic level with the MAL-collagen pathway and provides evidence that Fn14 itself is regulated by ET-1.

It has previously been shown that inhibition of Fn14 signaling reduces fibrotic processes in several organs in disease models [19, 51, 53, 54]. In the heart, this pathway has been associated with LV remodeling. Systemic overexpression of TWEAK induced heart failure affecting LV and RV [20]. However, it remained unclear whether endogenous TWEAK/Fn14 signaling plays an active role in cardiac remodeling. Decreased TWEAK blood levels in patients with PAH argue against a role in the RV [11]. However, reduced TWEAK blood levels might be due to sequestration of circulating TWEAK by the upregulated Fn14 receptor or might be a compensatory mechanism to protect from the consequences of Fn14 activation. This is supported by our findings that TWEAK blood plasma levels were reduced in MCT-treated rats while Fn14 was upregulated in the heart. In fact, opposite to most other signaling principles, in vivo TWEAK/FN14 pathway activation is regulated by changing the concentration of the receptor, not of the ligand, [1]. Moreover, high Fn14 levels are reported to activate downstream signaling with few or none TWEAK present [50]. Finally, correlations of cytokine blood levels to heart disease can be misleading as exemplified by TNF [27]. Interestingly, TWEAK blood plasma levels are also decreased in patients and animal models with CKD whereas Fn14 was upregulated in the kidney. Importantly, deletion of Fn14 in the animal models protects against kidney fibrosis and failure [19, 52].

Fibrosis is characterized by fibroblast accumulation and excess deposition of extracellular matrix proteins, which leads to tissue remodeling and dysfunction. The traditional view is that the underlying mechanism is induction of resident fibroblast proliferation [24]. Our data suggest that reduced RV fibrosis upon PAB in Fn14^−/−^ mice is partially due to inhibition of this process. This is in accordance with reports that TWEAK has pro-mitogenic effects on cardiac cells including fibroblasts [5, 28, 35]. Thus, reduced numbers of myofibroblasts and fibrosis might be due to reduced fibroblast proliferation. However, as the activation of TWEAK/Fn14 signaling was sufficient to induce myofibroblast differentiation and collagen expression in vitro, it appears likely that induced Fn14 re-expression also promotes myofibroblast differentiation driving RV fibrosis.

The traditional view of fibrosis has been challenged during the last years as it became clear that the fibroblast population exhibits a large phenotypic heterogeneity [24]. It is now clear that fibroblasts can be derived from endothelial cells, pericytes, bone marrow-derived progenitor cells, monocytes, and fibrocytes. Thus, it is possible that inhibition of TWEAK/Fn14 signaling is not only regulating fibroblasts proliferation and collagen expression but also fibroblast precursor recruitment. Whether this plays in PAB-induced fibrosis a major role is unknown. However, it is well known that the TWEAK/Fn14 axis affects the immune response upon tissue injury [4] and it has been hypothesized that it induces the recruiting of proinflammatory mediators during the acute phase of MI while at later time points, it participates in extracellular matrix remodeling and fibrosis. Therefore, it will be interesting to determine in future experiments whether TWEAK/Fn14 controls fibroblast precursor recruitment in the PAB model.

Although our in vitro data indicate that TWEAK can modulate directly fibroblast proliferation, differentiation and collagen synthesis, we cannot conclude from our data whether the observed protection is due to a direct myocardial effect or due to an unknown systemic, e.g. immune-mediated effect as we have utilized a general knockout model. Future studies utilizing conditional knockout mice will elucidate the underlying cellular mechanism.

*SM22 and SMA* both contain CArG boxes in their transcription control region, which are known targets of actin-MAL/MRTF-SRF signaling [9, 44, 49]. MAL directly regulates *Col1a2* gene expression [44]. It is a downstream target of Rho GTPase-actin signaling, which targets ROCK and MLC kinases [32]. Nuclear MAL translocation links reorganization of the actin cytoskeleton to SRF-dependent gene transcription and myofibroblast differentiation [16, 25, 44]. Finally, ROCK inhibition can reduce cardiac fibrosis [16, 17, 40]. In accordance with these data, TWEAK stimulation led to rapid activation of RhoA kinase in CFs. Blockage of the Rho/SRF or ROCK activity abolished nuclear MAL translocation and reduced collagen expression. Our data suggest that TWEAK activates via Fn14 the RhoA-ROCK-dependent nuclear translocation of MAL to trigger SRF-dependent transcription. As Chen and co-workers have recently demonstrated that TWEAK/Fn14 signaling promotes proliferation and collagen synthesis of rat CFs via the NF-кB pathway it will be interesting to determine in the future how these pathways interact [5, 48, 55].

Fn14 expression itself can be induced in CFs by ET-1, one of the key regulators implicated in the pathogenesis of PAH [45]. Importantly, ET-1 also facilitated TWEAK/Fn14 signaling towards MAL translocation. Reduced ET-1 levels in Fn14^−/−^ mice are probably due to reduced fibrosis [10, 29].

RV failure is the leading cause of death in PAH [8, 38]. However, available therapies all target pulmonary vasoconstriction, but not the remodeling of the right heart [12]. Fn14 combines several features that make it a good therapeutic target: Fn14^−/−^ mice are viable and show no obvious phenotype under physiological conditions. Fn14 is specifically upregulated in the RV after PAB, and its genetic ablation protected from right heart disease. Thus, global inhibition during therapy appears not prone to cause side effects. However, it is possible that Fn14^−/−^ mice activate compensatory pathways. In that case, therapeutic inhibition might have negative effects. For example, it has recently been suggested, in contrast to previous reports, that the human heart is a highly dynamic organ with the ability to regenerate cardiomyocytes [22]. Thus, as TWEAK has recently been shown to be a positive regulator of cardiomyocytes proliferation [35], a therapy targeting TWEAK/Fn14 signaling might interfere with cardiac homeostasis and cause over adverse effect.

Anti-TWEAK blocking antibodies and Fn14-Fc decoy receptor are available and have successfully been tested in other disease models. Given our findings, blocking the TWEAK/Fn14 axis may be a useful therapy for protecting patients from right heart disease and therefore, warrants further preclinical investigation.

## Electronic supplementary material

Below is the link to the electronic supplementary material.

Supplementary material 1 (DOCX 49 kb)

Supplementary material 2 (DOC 48 kb)

Supplementary material 3 (DOC 31.5 kb)
