# Supplementary material for: Deletion of Fn14 receptor protects from right heart fibrosis and dysfunction
Source: Basic Res Cardiol. 2013 Jan 17;108(2):325. doi: 10.1007/s00395-012-0325-x (PMC3597271; doi:10.1007/s00395-012-0325-x)
Supplement: Supplementary file 1 — Supplementary material 1 (DOCX 49 kb) [file 395_2012_325_MOESM1_ESM.docx]

**Table S1**

|  | Pre-OP parameters | | | | |
| --- | --- | --- | --- | --- | --- |
|  | **SHAM** | | | **PAB** | |
|  | **Fn14^+/+^** | **Fn14^-/-^** | | **Fn14^+/+^** | **Fn14^-/-^** |
| group size | **n=5** | **n=4** | | **n=11** | **n=12** |
| RV EDV ^1^ | 37.75 ± 2.53 | 35.78 ± 3.39 | | 41.53 ± 1.93 | 40.19 ± 2.23 |
| RV ESV ^1^ | 22.01 ± 1.83 | 20.77 ± 3.29 | | 24.13 ± 1.48 | 21.74 ± 1.39 |
| RV EF [%] ^1^ | 41.90 ± 1.24 | 44.53 ± 5.07 | | 42.18 ± 1.54 | 42.90 ± 1.56 |
|  | **Post-OP parameters** | | | | |
|  | **SHAM** | | **PAB** | | |
|  | **Fn14^+/+^** | **Fn14^-/-^** | **Fn14^+/+^** | | **Fn14^-/-^** |
| group size | **n=5** | **n=4** | **n=11** | | **n=11** |
| RV EDV ^1^ | 38.32±3.19 | 35.84±4.153 | 64.70±4.56* | | 54.13±4.56* |
| RV ESV ^1^ | 23.38±2.177 | 21.52±2.531 | 49.89±3.12* | | 37.48±4.67* |
| RV EF [%]^1^ | 40.79±3.19 | 39.77±3.17 | 23.12±1.362* | | 32.72±3.38*, ^†^ |
| BW [g] | 24.48 ± 1.94 | 22.65 ± 1.39 | 24.64 ± 1.12 | | 22.48 ± 0.82 |
| RW [g] | 0.02 ± 0.002 | 0.02 ± 0.002 | 0.04 ± 0.002*** | | 0.035 ± 0.002** |
| RW/BW [mg/g] | 0.85 ± 0.03 | 0.85 ± 0.043 | 1.63 ± 0.083*** | | 1.59 ± 0.104*** |
| RW/TL [mg/mm] | 1.22 ± 0.11 | 1.16 ± 0.07 | 2.35 ± 0.15*** | | 2.09 ± 0.13** |
| RV/LV+S | 0.24 ± 0.09 | 0.24 ± 0.01 | 0.50 ± 0.02*** | | 0.47 ± 0.03** |
| LV [g] | 0.088 ± 0.006 | 0.079 ± 0.0053 | 0.079 ± 0.0028 | | 0.075 ± 0.0017 |
| LV/BW | 3.59 ± 0.074 | 3.48 ± 0.072 | 3.26 ± 0.102 | | 3.38 ± 0.076 |
| LV/TL | 5.11 ± 0.365 | 4.75 ± 0.231 | 4.68 ± 0.164 | | 4.45 ± 0.097 |
| HR [beats/min] ^1^ | 462.4 ± 13.45 | 441.5 ± 14.69 | 450.7 ± 14.35 | | 455.0 ± 13.35 |
| Systemic systolic Pressure [mm/Hg] ^2^ | 94.46 ± 1.52 | 100 ± 4.74 | 80.3 ± 2.98 | | 88.28 ± 3.01 |
| RV sys Pressure [mmHg]^3^ | 27.6 | 25.7 | 61.2 | | 64.0 |
| RV dp/dt Max [mm/Hg/s]^3^ | 1537 ± 127.2 | 1514 ± 116.2 | 3670 ± 331.9 | | 3717 ± 330.5 |
| RV dp/dt Min [mm/Hg/s] ^3^ | -1396 ± 100.7 | -1184 ± 109.1 | -3105 ± 311.2*** | | -3238 ± 233.4** |
| RV SV [µl] ^1^ | 14.94 ± 1.71 | 14.32 ± 2.13 | 15.27 ± 0.98 | | 17.61 ± 1.07 |
| CO [ml/min] ^1^ | 8.1 ± 0.88 | 6.8 ± 0.99 | - 1. 0.67 | | 7.9 ± 0.84 |

^1^ -MRI measurements

^2^- Right carotid artery

^3^- RV catheterization

**P* <0 .05; **P<0 .001; ***P<0 .001 - versus baseline

†*P* < .05 - versus Fn14**^+/+^**.
