# Supplementary material for: Deletion of Fn14 receptor protects from right heart fibrosis and dysfunction
Source: Basic Res Cardiol. 2013 Jan 17;108(2):325. doi: 10.1007/s00395-012-0325-x (PMC3597271; doi:10.1007/s00395-012-0325-x)
Supplement: Supplementary file 2 — Supplementary material 2 (DOC 48 kb) [file 395_2012_325_MOESM2_ESM.doc]

**Expanded Methods**

**Pulmonary artery banding**

After baseline MRI, mice were randomly assigned to 2 groups: SHAM-operated animals and mice subjected to PAB. PAB (n=22) and/or SHAM operation (n=9) was performed under isoflurane anesthesia (1.5% vol/vol) and a subcutaneous administration of 0.03 mg/kg buprenorphine hydrochloride. Mice were intubated and respiration was controlled by a rodent ventilator (Harvard Apparatus, USA). The left thorax was opened at the third intercostal space to expose the pulmonary artery, which was carefully dissected free from the ascending aorta, and a surgical hemoclip was positioned leaving the pulmonary artery constricted to a diameter of 0.35 mm. The thorax was then closed with a vicryl suture. SHAM-operated animals underwent the same surgical procedure except for the vessel banding.

**MRI measurements**

Cardiac MRI was performed before OP and 3 weeks post-OP. Cardiac MRI measurements were performed on a 7.0 T Bruker Pharmascan, equipped with a 300mT/m gradient system, using a custom-built circularly polarized birdcage resonator and the IntraGate^TM^ self-gating tool. MRI data were analyzed using Qmass digital imaging software (Medis). The 12-14 weeks old mice were measured under volatile isoflurane (1.5 – 2.0 %) anesthesia. The measurement is based on the gradient echo method (repetition time = 6.2 ms; echo time = 6.0 ms; field of view = 2.20x2.20 cm; slice thickness = 1.0 mm; matrix = 128 x 128; repetitions = 100). The imaging plane was localized using scout images showing the 2-and 4-chamber view of the heart, followed by acquisition in short axis view, orthogonal on the septum in both scouts. Multiple contiguous short-axis slices consisting of 7 to 10 slices were acquired for complete coverage of the left and right ventricle. Bodyweight changes and survival were monitored from 0 to 21 days for all experimental groups.

**Hemodynamic Assessment**

On day 21 after PAB mice were anaesthetized by inhalation of isoflurane 1.5% vol/vol. Core body temperature was maintained at 37°C using a controlled heating pad. A Millar microtip catheter (SPR-671, FMI, Foehr Medial Instruments GmbH, Seeheim/Ober-Beerbach, Germany) was inserted through the right jugular vein into the RV for measurement of RV pressure. Afterwards the same catheter was inserted into the left carotic artery to measure systemic arterial pressure. All hemodynamic measurements were performed with a PowerLab System using the Chart 7.0 Software (ADInstruments GmbH, Spechbach, Germany).

**Blood collection and TWEAK ELISA**

Blood of PAB and SHAM-operated mice (at 3 weeks) and MCT-treated rats (at 5 weeks) was intracardially collected and analyzed with a RayBio_Mouse TWEAK ELISA Kit.

**Primary Mouse Cardiac Fibroblast isolation**

Isolation of CFs were performed either by perfusion of the whole heart or by mincing ventricles in small pieces and serial digestion using the Liberase (TH) enzyme mix (Roche) (0.25mg/ml per 4 hearts). After complete digestion cells were collected in Horse Serum (HS) and centrifuged at 330 x g for 5 min. Two hours after seeding cells were washed.

**Cell culture experiments**

Rat2 fibroblasts cultured in medium (DMEM/F12) plus 10% FBS. For collagen assays cells were split in serum-free conditions and next day treated with ITEM2 and Mouse IgA (10 ng/ml, eBioscience) for 1 h prior TWEAK stimulation (48 h, 100 ng/ml, Biolegend). For cell count experiments Rat2 and CFs cells were split in serum-free medium. HEK293T and NIH3T3 cells were cultured overnight in serum-free DMEM medium with GlutaMAX^TM^ prior treatment. NIH3T3 cells were stimulated either with TWEAK or 15% FCS for 4 h prior fixation. Adult CFs were cultured in DMEM (Low Glucose medium, 1 g/L) before stimulation. All media contained 100 U/ml penicillin and 100 μg/ml streptomycin.

**Primer sequences**

The primers that were used were as follows: Col1a2: F: 5’-TTGGCCCATCTGGTAAAGA-3’, R: 5’-CAGGGAATCCGATGTTGCC-3’; Col3a1: F: 5’-TGGTCCTCAGGGTGTAAAGG-3’, R: 5’-GTCCAGCATCACCTTTTGGT-3’; Col4a1: F: 5’-CTCCCTGGGACTCCTGGCCC-3’, R: 5’-TTCTCTCCTGCCCGACCCCGG-3’; mANP: F: 5’-AGATGAGGTCATGCCCCCGCAG-3’; R: 5’-GCGAGCAGAGCCCTCAGTTTGC-3’; mMyh7: F: 5’-ACCGGAGAATCCGGAGCTGGT-3’; R: 5’-CAAGGTTGCCCTTGCCTGGGG-3’; mSM22: F: 5’-CACTGCCTAGGCGGCCTTTAAACC-3’, R: 5’-GCACTTCTCGGCCTCATGCCG-3’; mSMA: F: 5’-TCAGGAACCCTGAGACGCTGCT-3’; R: 5’-CCAGCGAAGCCGGCCTTACAG-3’; GAPDH: F: 5’-AACTTTGGCATTGTGGAAGG-3’; R: 5’-ACACATTGGGGGGTAGGAACA-3’; mFn14 (Ex1-2 Junction) F: gattcggcttggtgttgatg, R: cagtccatgcacttgtcgag, mFn14 (Ex2-3 Junction) F: gacctcgacaagtgcatgg, R: ggccacagtagcctgaagtg; mFn14 (Ex3-4 Junction) F: gctggtttctagtttcctggtc, R: actggatcagtgccacacct; m,r ET-1 F: AGAAACAGCTGTCTTGGGAGC, R: ACACACTCCTTGTCCATCAAGG.

**Construction of pEGFP-TWEAK**

PCR was performed using cDNA from HEK293T cells, HotStar HiFidelity Polymerase (QIAGEN) and following primers: sense 5'-ATGGCCGCCCGTCGG-3' and antisense 5'-TCAGTGAACCTGGAAGAGTCCG-3'. The DNA product was ligated into pGEM-T-easy (Promega). A HindIII/SalI TWEAK fragment was subcloned into pEGFP-N1 (primers: sense 5'-GGCGAAGCTTATGGCCGCCCGTCGG-3' and antisense 5'-CCGCGTCGACCAGTGAACCTGGAAG-3').

**Transfection and luciferase promoter assays**

Transient transfections of HEK293T cells (5 x 10^3^ cells per transfection, 50% confluence in 96-well plates) were performed in serum-free medium with Fugene 6 (Roche) with a total of 50 ng of constructs. Col1a1, Col1a2 and EmptyPromoter and RandomPromoter luciferase GoClone reporter constructs were purchased from SwitchGear Genomics. pEGFP-N1 (Clontech), pEGFP-TWEAK (25 ng), pEF-MAL-HA (FL) (5 ng) were co-transfected together with the reporter constructs. Normalization was performed with the EmptyControl (SwitchGear Genomics). Luciferase activity was measured by LightSwitch Luciferase Assay Reagent (Mitras LB 940 Luminometer, Berthold Technologies) after 24 h of stimulation or 30 to 48 h after overexpression.

**Western Blotting**

Tissues were lysed and homogenized in RIPA buffer containing 1 mM EDTA and 1x Protease Inhibitor Cocktail (Thermo Scientific). Homogenization of the lysates was performed in screw cap tubes containing ceramic lysis beads at 6000 rpm, 90 s, 2 cycles at 4°C in Precellys®24 (PeqLab). After 90 s of homogenization the samples were centrifuged at 17000 x g at 4°C for 30 min. The protein concentration was determined using Bio-Rad DC Protein Assay according to manufacturer protocol (Bio-Rad). Equal amount of proteins (30 µg) were resolved by 12% Novex Bis-Tris Gels (Invitrogen) and blotted on nitrocellulose membranes (Protran®). Membranes were blocked with 5% non-fat dry milk in Tris-buffered saline (TBS, 10 mM Tris-HCl (pH 7.5), 150 mM NaCl) with 0.1% Tween for 1 h at RT and incubated with primary antibodies diluted in 5% milk/TBS/T and/or 5%BSA/TBS/T over night at 4°C: rabbit anti-TWEAK/Fn14 receptor, rabbit anti-pan-actin, monoclonal rabbit anti-RhoA (1:1000) (all Cell Signaling), rabbit anti-Collagen Type 1 (1:500) (Rockland Immunochemicals), mouse anti-GAPDH (1:2000) (Sigma). Antigen-antibody complexes were visualized using horseradish peroxidase-conjugated secondary antibodies (Amersham) and SuperSignal ® ECL detection system (BioRad).

**Determination of activated RhoA**

Cells were lysed in 50 mM Tris, pH 7.2, 1% Triton X-100, 0.5% sodium deoxycholate, 0.1% SDS, 500 mM NaCl, 10 mM MgCl2, 10 µg/ml each of leupeptin and aprotinin, and 1 mM phenylmethylsulfonyl fluoride, and clarified cell lysates were incubated with GST-RBD at 4°C for 50 min. Bead pellets were washed and finally resuspended in 25 µl of Laemmli sample buffer. Proteins were separated by SDS-PAGE and transferred to nitrocellulose membrane and probed for RhoA using a specific antibody.

**Immunohistochemistry**

For immunohistochemistry on paraffin sections mouse hearts were isolated, dissected in RV and LV+S, washed in cold PBS, fixated in 10% PFA for overnight, embedded in paraffin and RVs were sectioned longitudinally (5 µm). Sections were deparaffinized in xylene and rehydrated in ethanol. Heat–mediated antigen retrieval was performed in 0.05M EDTA buffer (pH 8.0). For determination of Fn14, TWEAK and P4HB protein expression sections were stained with rabbit polyclonal anti-Fn14 antibody at a dilution of 1:100 (Cell Signaling), rabbit polyclonal anti-TWEAK antibody (1:100) (Abcam) and mouse monoclonal anti-P4HB (1:20) (Acris) antibodies with subsequent detection using mouse AP-conjugated IgG and BM Purple AP substrate (Roche). The peroxidase-conjugated Dako REAL EnVisionDetection System, Peroxidase/DAB+-kit (Dako) was used to detect the Fn14 and TWEAK antibodies. For counterstaining of nuclei Methyl Green was used. For Sircol assay RV sections sections were first deparaffinized at 58°C for 1 h. Staining was performed in 0.1% Sirius Red F3B in picric acid for 1 h at RT. After short washing in 1% glacial acetic acid sections were dehydrated and cleared in xylene. For immunohistochemistry on cryosections mouse hearts were dissected in RV and LV+S, washed in cold PBS, frozen in cryo-embedding media (OCT) and cut in 5 µM thickness sections. After 5 min of acetone fixation sections were washed with PBS and stained with goat anti-DDR2 antibody (1:100), rabbit anti-Fn14 antibody (1:100) and mouse alpha-actinin (1:100) for overnight at 4°C.

**Quantitative analysis of fibrosis**

To detect collagen fibers RV sections of SHAM- and PAB-operated mice were stained with 0.1% Sirius red F3B in picric acid. Images were taken using a 40x objective. Photomicrographs of RV sections were analyzed to determine the interstitial collagen fraction using a QWin V3 computer-assisted image analysis software (Leica, Microsystem, Wetzlar, Germany). After correction of background and setting the threshold, the software sequentially opened each image, performed the analysis of a number of fields in a serpentine fashion (from one end of the tissue moving toward the other), stored the data and closed the image. Obtained data represent average results from 5 Fn14^+/+^ and 7 Fn14^-/-^ hearts (≥ 19 sections and ≥ 240 fields per heart).

**Immunofluorescence**

Heart sections were blocked in 5% goat serum/0.2% Tween-20/PBS for 1 h at RT. NIH3T3 cells were preincubated for 1 hour with ROCK kinase inhibitor Y27632 (20 µM) and Rho-SRF pathway inhibitor CCG-1432 (5 µM) prior TWEAK stimulation. NIH3T3 and Rat2 cells were fixated for 15 min in 3.7% paraformaldehyde and permeabilized for 5 and 10 min respectively in PBS/0.5% Triton. CFs were fixated in 3.7% paraformaldehyde and permeabilized for 5 min in PBS/0.5% Triton. Primary antibodies were diluted in appropriate blocking solution and incubated at 4°C for overnight. For DDR2 stainings cells were blocked with 1% BSA buffer and subsequently stained with goat anti-DDR2 antibody (1:200). For P4HB and MAL stainings cardiac fibroblasts were blocked in 5% goat serum/0.2% Tween-20/PBS. The following antibodies have been used for immunofluorescence staining: mouse alpha-actinin (1:50, Abcam), rabbit anti-PCNA (1:50, FL-261, Santa-Cruz), mouse anti-MAL (clone 1A11) (1:50), mouse smooth muscle actin (1:100, Sigma), mouse anti-Caveolin 3 (1:100, BD Transduction Laboratories), goat anti-DDR2 (N-20, 1:200, Santa-Cruz), rabbit anti-P4HB (1:100, Abcam). Primary antibodies were detected with goat anti-rabbit/anti-mouse Alexa Fluor 488/594 (1:200, Invitrogen). Nuclei were visualized by DAPI. F-actin was detected by rhodamine-phalloidin and cell membranes by WGA (Wheat Germ Agglutinin) staining (Molecular Probes, Invitrogen). For the quantitative analysis of PCNA-positive interstitial cells after PAB 14-20 random fields at a 40x magnification at 3 different levels of each RV were analysed. Quantification of interstitial cell numbers were performed on paraffin sections using mouse alpha-actinin and DAPI and ImageJ software (21 sections per group, mean ± SEM, ≥ 3 random fields at x10 magnification at 3 different levels). Cardiomyocyte size was determined by staining with mouse anti-Caveolin antibody. Measuring of the cell size was performed using the ImageJ software (50 cells per section, 4 random sections per heart).

**Endothelin stimulation**

After serum starvation cells were stimulated for 24 h with 100 nM ET-1 (R&D Systems). Subsequently, TWEAK (100 ng/ml) was added. MAL translocation was determined after 8 hours.

**Collagen assay**

Serum-starved Rat2 fibroblasts were stimulated with TWEAK (100 ng/ml) for 48 hours. L-ascorbic acid (0.25 mM) was added to the medium daily. Cells were lysed in RIPA buffer and total collagen (Types 1-5) was assessed using a Sircol soluble collagen assay kit (Biocolor Ltd). Collagen was measured by reference to a type-I collagen standard curve.

**MAL translocation**

To determine MAL translocation the Count software (by B. Waclaw) was utilized. The nuclei number was determined based on DAPI staining. MAL translocation was considered positive if the pixel number in the purple channel (MAL/DAPI overlap) was greater or equal compared to the threshold defined by the control (unstimulated) experiment.

**Proliferation assay**

Proliferation was determined with a CountessTM cell counter (Invitrogen) or CellTiter 96 Aqueous Cell Proliferation Assay (Promega). Rat2 fibroblasts were seeded in 35-mm plates at the density of 1 x 10^5^ and kept for overnight in serum-free medium. Serum-starved Rat2 fibroblasts were stimulated for 48 hours with increasing concentrations of TWEAK as indicated. Viability and cell count measurements were performed using the trypan blue method. Rat2 fibroblasts were infected 24 h after plating in serum-deprived media (0.2%) with AdFn14 or Adβ-gal control (50 MOI) for 48 h. After infection with adenoviruses the proliferation of cells were detected for colorimetric detection of colored formazan that is bioreduced by cells from MTS tetrazolium compound (CellTiter 96 Aqueous Cell Proliferation Assay, Promega) according to the manufacturer’s protocol.
