# Supplementary material for: Deletion of Fn14 receptor protects from right heart fibrosis and dysfunction
Source: Basic Res Cardiol. 2013 Jan 17;108(2):325. doi: 10.1007/s00395-012-0325-x (PMC3597271; doi:10.1007/s00395-012-0325-x)
Supplement: Supplementary file 3 — Supplementary material 3 (DOC 31.5 kb) [file 395_2012_325_MOESM3_ESM.doc]

**Supplemental figure legends**

**Figure 1.** Characterization of TWEAK and Fn14 levels. (*A*) Immunohistochemistry showed that TWEAK protein expression is not significantly changed after PAB in RV of Fn14^+/+^ animals. Nuclei were counterstained with Methyl Green. (B) TWEAK blood plasma levels of animals 3 weeks post PAB. (*C* and *D*) Fn14 and TWEAK expression of RV and LV following monocrotaline treatment (MCT) were analysed by Real-Time PCR analysis. *gapdh* was used as loading control (*p < 0.05, means ± SEM, n > 4). RV: right ventricle. LV: left ventricle. (E) TWEAK blood plasma levels of animals after 5 weeks of MCT treatment. TWEAK levels were significantly reduced in MCT-treated animals (*p < 0.05, means ± SEM).

**Figure 2.** Analysis of Fn14 Expression in cultured cells and collagen expression. (*A*) Western blot analyses of endogenous Fn14 expression in HEK293T, NIH3T3 and Rat2 fibroblasts. (*B* and *C*) Sircol assays confirming TWEAK-induced collagen expression in Rat2 fibroblasts. (*B*) The collagen expression level of untreated cells was set to 100% (mean ± SEM, n = 4, **p < 0.005). (*C*) To test whether Fn14 signalling is required for TWEAK-induced collagen synthesis we employed the Fn14 blocking antibody ITEM2. TWEAK-induced synthesis of collagens was significantly reduced in the presence of ITEM2 (mean ± SEM, n = 3, *p < 0.01). (*D* and *E*) To test whether MAL is required for TWEAK-induced collagen synthesis we employed siRNA against MAL. As control we utilized scrambled siRNA. TWEAK-induced synthesis of collagens was abolished in the presence of MAL siRNA (mean ± SEM, n = 4, *p < 0.05).

**Figure 3.** Fn14 regulates myofibroblast differentiation and MAL translocation. (*A*) TWEAK induced nuclear translocation of MAL in NIH3T3 cells. Scale bars: 50 µm. (*B*) Quantitative analysis of MAL translocation. (*C*) HEK293T cells were stimulated with TWEAK and the amount of activated RhoA was determined by immunoprecipitation with Rho-binding domain of Rhotekin (GST-RBD). (*D*) Immunofluorescence analyses of actin fibers (Rhodamine-phalloidin, red). TWEAK stimulation resulted in the enrichment of actin fibers in Rat2 fibroblasts. Scale bars: 50 µm.

**Figure 4.** Fn14 regulates fibroblasts proliferation. (*A*) Immunofluorescence analysis with fibroblast-specific markers (DDR2, P4HB) indicating that the isolated primary non-myocytes are to > 90% fibroblasts. (*B*) TWEAK increased the cell number of Rat2 fibroblasts in a dosage-dependent manner (mean ± SEM, n = 5, *p < 0.05, **p < 0.001). (*C*) Western blot analyses of adenoviral mediated Fn14 protein overexpression in Rat2 fibroblasts. Equal amounts of protein extracts (35 µg) were loaded and pan-actin was used as loading control. (*D*) Colorimetric proliferation assay demonstrating the pro-proliferative effect of Fn14. Rat2 cardiac fibroblasts were infected with AdFn14 or Adβ-Gal (mean ± SEM, n = 5, **p < 0.005, ***p < 0.0001). Data are represented as absorbance relative to control cells.
